# Resensitization to colistin results in rapid and stable recovery of adherence, serum resistance and ompW in *Acinetobacter baumannii*

**DOI:** 10.1371/journal.pone.0309307

**Published:** 2024-08-28

**Authors:** Jale Boral, Cansel Vatansever, Gulin Ozcan, Siran Keske, Sirin Menekse, Mehmet Gonen, Fusun Can

**Affiliations:** 1 Graduate School of Health Sciences, Koç University, Istanbul, Türkiye; 2 Koç University İşBank Center for Infectious Diseases (KUISCID), Koç University Hospital, Istanbul, Türkiye; 3 Department of Medicine, Division of Gastroenterology and Hepatology, University of Illinois at Chicago, Chicago, Illinois, United States of America; 4 Department of Infectious Diseases, Koşuyolu Kartal Heart Training and Research Hospital, İstanbul, Türkiye; 5 Department of Industrial Engineering, College of Engineering, Koç University, Istanbul, Türkiye; Nitte University, INDIA

## Abstract

**Background:**

Colistin resistance in *Acinetobacter baumannii* is an emerging problem that limits antimicrobial therapy options.

**Materials & methods:**

We isolated two pairs of colistin susceptible and colistin-resistant *A*. *baumannii* (K1007/K1006 and K408/K409) from two patients diagnosed with carbapenem-resistant *A*. *baumannii* infection. Colistin susceptible isolates were exposed to *in vitro* colistin induction for 50 generations. The selected cell populations were subjected to DNA and RNA sequencing and phenotypic assays.

**Results:**

In the *in vitro* induction assay, K408 gained colistin resistance on the corresponding day of clinical resistance (K408-G25) and got resensitized to colistin in the consecutive generation (K408-G26). A significant upregulation of ompW, *ata*, *adeFGH* genes on K408-G25 was followed by a downregulation upon resensitization to colistin (G26). Despite the upregulation of the *ompW* gene in transcriptomic analysis, the ompW protein disappeared on K408-G25 and recovered in the resensitized generation (G26). In parallel, disrupted cell membrane integrity recovered in K408-G26. In the K408-G25, downregulation of *pbpG and* upregulation of *pbp1a/pbp3* genes decreased serum-resistance which was reversed in the resensitized generation (G26). The K1007 did not gain colistin resistance amongst 50-generations, however, the generation corresponding to clinical resistance day (K1007-G9) had a similar trend with K408-G25. The clinical colistin-resistant K409 and K1006 had SNPs on *pmrA* and *pmrB* genes.

**Conclusion:**

In this study, we observed that *A*. *baumannii* regulates adhesion, efflux pumps and serum-resistance associated genes as an early response to colistin stress. Besides, the ompW protein disappears in the cell membrane of colistin resistant cells which recovers after resensitization to colistin. The lack of ompW protein in colistin-resistant cells should be taken into consideration for escape mutants in development of antivirulence vaccination or treatment options.

## Introduction

Emerging antimicrobial resistance is one of the major problems that makes *Acinetobacter baumannii* a pathogen of concern both for community acquired and hospital acquired infections [1,2]. Globally, carbapenem resistance rate in *A*. *baumannii* is reported over 90% with a higher prevalence in Mediterranean region while colistin resistance rate fluctuates in a lower range between 0.9–3.3% [3–7].

*The pmrA*, *pmrB*, *pmrC*, *lpxC and lpxD* mutations are known to be linked with colistin resistance by entailing the loss of LPS leading to further modifications in virulence [8–10]. Diminishing virulence scores amongst *lpx* mutant colistin-resistant *A*.*baumannii* compared to colistin-susceptible isolates was previously reported [11]. The virulence factors of *A*. *baumannii* such as capsule formation, biofilm formation, adherence, surface motility, serum resistance, effective use of secretion mechanisms, RND efflux pumps and outer membrane proteins are known to contribute to high tissue invasion, thus, result in enhanced pathogenesis [12,13]. The colistin resistance has been associated with reduced surface motility, decreased biofilm production, and reduced *in vivo* dissemination ability [14]. However, high infection (41.9%) and fatality rates (100%) are being reported for colistin-resistant *A*. *baumannii* [15]. Given the high case fatality rates, understanding the adaptation mechanisms to colistin resistance remains important to illuminate the underlying mechanisms for the pathogenesis and progress of the disease.

Colistin resistance mechanisms and interchanging virulence factors were previously studied using *in vitro* colistin induction assays; however, *in vitro* colistin stress does not completely reflect the outcome of colistin stress in isolates from patients receiving colistin therapy [16,17]. Therefore, clinical susceptible and resistant isolates obtained from patients under colistin therapy should be longitudinally studied.

In this study, we comparatively studied two pairs of colistin susceptible and resistant isolates from two patients. The isolates and their subcultures on colistin-containing media for 50 generations were analysed on, phenotypic, metagenomic and transcriptomic basis regarding their interchanging virulence profiles during adaptation to prolonged colistin stress.

## Materials and methods

### Ethics statement

This retrospective study was approved by the Koç University Institutional Review Board (No: 2018.046.IRB2.009). The data access date is 24^th^ of May 2018. The data were anonymized and none of the authors in the team could identify individual participants during or after data collection.

### Patient Selection and in vitro colistin induction assay

**Fig 1:**
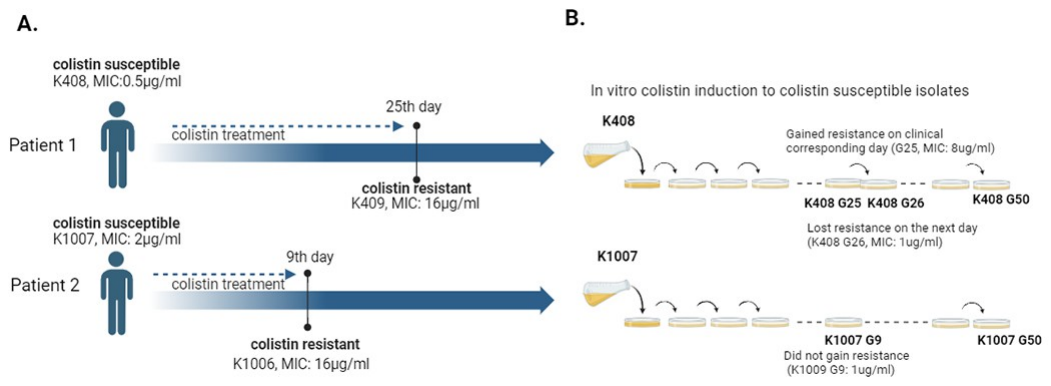
Schematic illustration of the study design (A) Sample selection based on colistin susceptibility and duration of colistin therapy. *In vitro* colistin induction (B) 4X MIC colistin induction to selected colistin-susceptible isolates (K408, K1007).

Two patients with isolations of colistin susceptible *A*. *baumannii* and colistin resistant *A*. *baumannii* after receiving colistin therapy were included in this study. The first colistin susceptible/colistin resistant *A*. *baumannii* pair (K408 = colS; K409 = colR) was isolated from wound infection and the second pair (K1007 = colS; K1006 = colR) was isolated from blood. The study design for sample selection and *in vitro* colistin induction was illustrated in Fig 1.

Colistin susceptible isolates were consecutively passaged onto Mueller Hinton (MH) medium which contains 4XMIC colistin and incubated overnight at 37°C. Unless there is no conferred *in vitro* colistin resistance, generational passages were pursued up to 50 passages (50 days). Colistin MIC values were checked for each generation using the microbroth dilution method.

Isolates were tested for their susceptibility to colistin using microbroth dilution method according to EUCAST guidelines [18]. Isolates were incubated overnight at 37°C in serially diluted colistin (Merck, USA) and the experiment was performed in quadruples.

### Molecular studies

Genomic DNA library preparation was done using Nextera DNA Library Prep Kit (Illumina, USA) according to the manufacturer’s instructions with no alterations. DNA isolation of samples was done using DNeasy UltraClean Microbial Kit QIAGEN, USA), based on manufacturer’s instructions and 400 ng DNA was adjusted by Qubit (Thermo-fisher, USA) with using Qubit dsDNA HS Assay Kits. Produced libraries were normalised based on their concentrations using QIASeq Library Quant Assay kit for the pooling of the samples. The pooled library was sequenced on a Novaseq (Illumina, USA) platform resulting in 150-bp paired-end reads and 50X coverage [19].

RNA isolation was done using RNeasy Kit (QIAGEN, USA) with no alterations to the manufacturer’s protocol. RNA library preparation for transcriptome sequencing was done using QIAseq^®^ Stranded RNA Library Kit (QIAGEN, USA). RNA samples were checked for their RNA integrity number (RIN) using Agilent RNA 6000 Nano Kit with no alterations on manufacturer’s protocol. RIN score above 8 was accepted as suitable for library preparation protocol. Qualitative assessment of prepared libraries was done using High Sensitivity DNA kit (Agilent 2100 Bioanalyzer, USA). Quantitative assessment of prepared libraries was done using QIASeq Library Quant Assay kit for the pooling of the samples. Pooled libraries were sequenced using Illumina NovaSeq platform resulting in 150-bp paired-end reads with 10 million reads per sample.

The Whole Genome Sequencing (WGS) data underwent quality control using FastQC (v0.11.9). Trimmomatic (v0.39) was utilised for quality trimming of raw reads. Burrows-Wheeler Aligner (BWA) (v0.7.17), employing the BWT algorithm, aligned the genome assembly to the reference sequence *Acinetobacter baumannii* ATCC 17978 with the NCBI Reference Sequence code NZ_CP033111.1. Sambamba (v0.7.1) was employed to remove duplicated reads. SAMTools (v1.13) was utilised to generate variant information through resequencing analysis using SAM/BAM files. Assembled sequences were annotated using SnpEff (v5.0e). Allelic profiles and whole genome sequence types (wgSTs) were determined using Applied Math Bionumerics V8.1 software by bioMerieux, employing default parameters.

The *Acinetobacter baumannii* genome (ASM1467277v1.) served as the reference genome for mapping 100 nt long RNA reads. Transcriptomic data was processed using Rsubread (v2.14.2) to obtain TPM (transcripts per million) gene expression values. Heat maps in this study depict log2(TPM + 1) expression levels of the genes. Gene ontology information was obtained from https://biocyc.org/.

Bioinformatics analyses were conducted using the R programming language. The genomic and transcriptomic data were deposited to https://midas.ku.edu.tr/Acinetobacter/.

The genes involving colistin resistance and genes associated with virulence mechanisms listed as pmrA, pmrB, pmrC, ata, ompA, adeA, adeB, adeC, adeF, adeG, adeH, adeI, adeJ, carO, were also analysed by qPCR. SYBR^®^ Green (Thermo Fisher, USA) qPCR master mix was used and cT values were diverted into fold values using ΔΔCt calculation [20]. Normalisation of Ct values was done using *A*. *baumannii* 17978 ATCC. List of primers are provided on S2 Table.

### Pulsed field gel electrophoresis

The isolates were assessed for their clonal relatedness using Pulsed-field gel electrophoresis (PFGE). The method and alterations used were taken from Boral et al [2]. Bionumerics 7.6 (Biomerieux, France) was used as the software to assess quantitative clonality. The similarity scores were indexed using the Dice Coefficient at a tolerance value of 1%.

### SDS-PAGE assay

Outer membrane protein isolation was performed from colonies grown to log phase in LB broth using a previously published protocol with some modifications [21]. Briefly, ultracentrifuge was used to pellet bacterial suspension at 110,000*g x* for 50 minutes at 4°C. Bradford assay was performed for the quantification of protein concentrations (Invitrogen, USA). SDS-PAGE assay was performed [22]. Imaging of the gel was done using ChemiDocX (Biorad, USA).

### Serum resistance assay

*A*.*baumannii* isolates were exposed to pooled normal human sera (NHS) for up to 3 hours by incubating at 37°C at 180 RPM based on King et al’s protocol [23]. Mixtures of normal human serum and bacterial suspension were transferred onto TSA at 0 minutes, 90 minutes and 180 minutes incubation. Inhibition rate of bacterial growth in the NHS was calculated using inactivated serum. *A*.*baumannii* ATCC 19798 was used as a control strain. This experiment was carried out in duplicates and repeated twice.

### Biofilm assay

Crystal violet staining assay was performed to assess biofilm formation ability [24]. Briefly, bacterial suspensions (1.5 × 107 CFU/ml) were added to a 96‐well flat‐bottom microtiter plate and cultured at 37°C for 24 hours. The cells were washed twice with distilled water and stained with 200 μl of 0.1% crystal violet (Sigma, St. Louis, MO, USA) for 30 minutes. The wells were then washed with PBS. After drying the plate at 60°C for 15 minutes, the stained biomass samples were dissolved in 95% ethanol. The OD540 reading for each well was determined using a Thermo MultiskanGo (Thermo, USA). All experiments were performed in triplicates.

### TEM electron microscopy imaging

Bacterial isolates were prepared for visualisation under Transmission Electron Microscopy to assess morphological changes upon colistin exposure. Bacterial cells were incubated at 37°C overnight, grown cells were pelleted then fixed in 0.1M phosphate buffer containing 3% glutaraldehyde and 1.5% paraformaldehyde for 2 h at 4°C [25]. Eventually, uranyl acetate was used for staining and HT7800 TEM (Hitachi) was used for visualisation.

### Statistical analysis

The statistical significance analysis of findings was done using IBM SPSS Statistics (Version 27). The independent samples t-test and and mann-whitney u test tests were performed for the analysis.

## Results

None of the patients received colistin before the isolation of colistin susceptible isolates (Patient 1:K408 and Patient 2: K1007). Colistin resistant pair of K408 was isolated on 25th day of colistin treatment (K409, MIC: 16ug/ml) and colistin resistant pair of K1007 was isolated on 9th day of colistin treatment (K1006, MIC: 16ug/ml). ColS (K1007, K408) and ColR (K1006, K409) isolates were clonally related to each other with similarity scores of >90% based on Tenover’s criteria [26] (S1 Fig).

**Fig 2:**
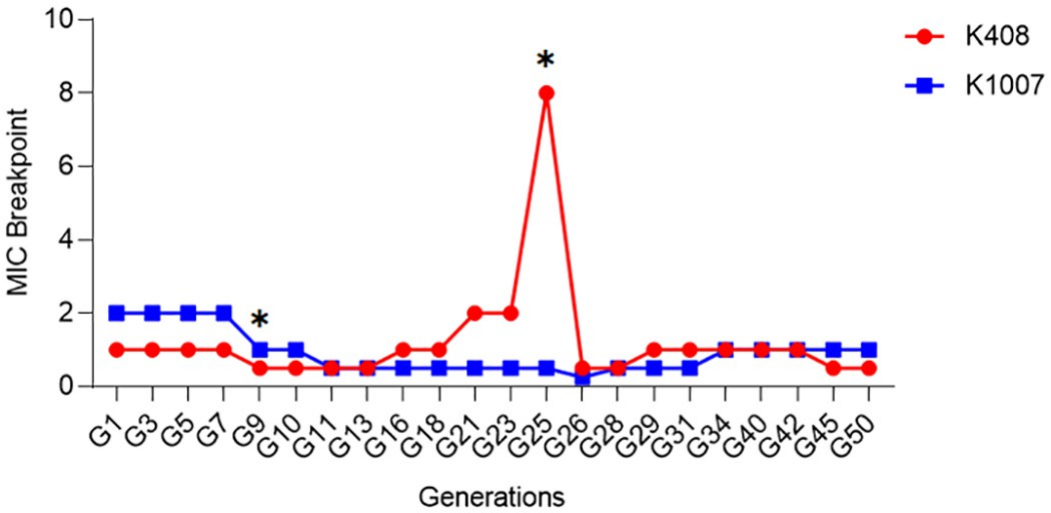
MIC breakpoint values of generations. MIC values amongst 50 generations for the sets of K408 and K1007 for colistin. *stars indicate the duration of colistin therapy received at the day of colistin resistant A. *baumannii isolation from patients 1 and 2*. *(K1007 G9; K408 G25)*.

In the *in vitro* colistin resistance induction assay, K408 gained resistance to colistin (MIC: 8ug/ml) on the 25th day of generational passaging (K408 G25) which was the corresponding clinical colistin resistance day. However, by 26th day of colistin exposure, the isolate got re-sensitised to colistin with a MIC of 1ug/ml (K408 G26) and has not gained colistin resistance amongst 50 days of generational passaging. On the other hand, K1007 did not gain resistance amongst 50 days of generational passages (K1007 G50). The changing MIC values upon colistin exposure were shown on Fig 2.

### Differences on virulence of paired colistin susceptible/resistant isolates and generations

In the transcriptomic dataset of isolates, all genes related to virulence traits, antimicrobial resistance, colistin resistance, metabolism, stress response and efflux systems were analysed and the genes that exhibit discrepant behavioural patterns were confirmed further using qRT-PCR and screened for SNPs. The heatmap of transcriptomic data was presented on S2 Fig.

Among all virulence and antimicrobial resistance related genes, discriminative SNPs were majorly observed at adhesion(*ata*), serum resistance (*ftsl*), quorum sensing (*abaI*) and colistin resistance (*pmrA*, *pmrB*) associated genes (Table 1).

**Table 1:** Single nucleotide polymorphisms in virulence and colistin resistance genes.

Function	Gene	Annotation Type	Codon Change	Protein Change	Isolate Codes
**Adhesion**	*ata*	SNP	4086T>A	As362Glu	K408 G25*
	*ata*	SNP	2597A>G	Asn866Ser	K408 G25*, K409*, K1007 G50*
	*ata*	SNP	2717C>T	Ala906Val	K408 G25*, K409*, K1007, K1007 G50
**Serum resistance**	*ftsI*	SNP	1544C>T	Ala515Val	K1006*
**Quorum Sensing**	*abaI*	SNP	359G>A	Ser120Asn	K1006*
**Colistin Resistance**	*pmrB*	SNP	412G>A	Ala138Thr	K1006*
	*pmrA*	SNP	36G>T	Met12Ile	K409*

*Represents colistin resistant isolates.

### Adherence

In colistin resistant generation of K408 (K408 G25), the *ata* gene associated with adherence and Type IV secretion system has exhibited SNPs on Asn866Ser and Ala906Val regions (Table 1). However, these substitutions recovered in the resensitized generation (K408 G26) and did not reappear amongst 50 generations. Besides, K408 G25 had As362Glu substitution which was not present in other generations. The same SNPs on Asn866Ser and Ala906Val regions were also detected in the clinical resistant isolate(K409). The non-discriminative mutual SNPs for cell sets of K408 and K1007 were shown in S1 Table.

**Fig 3:**
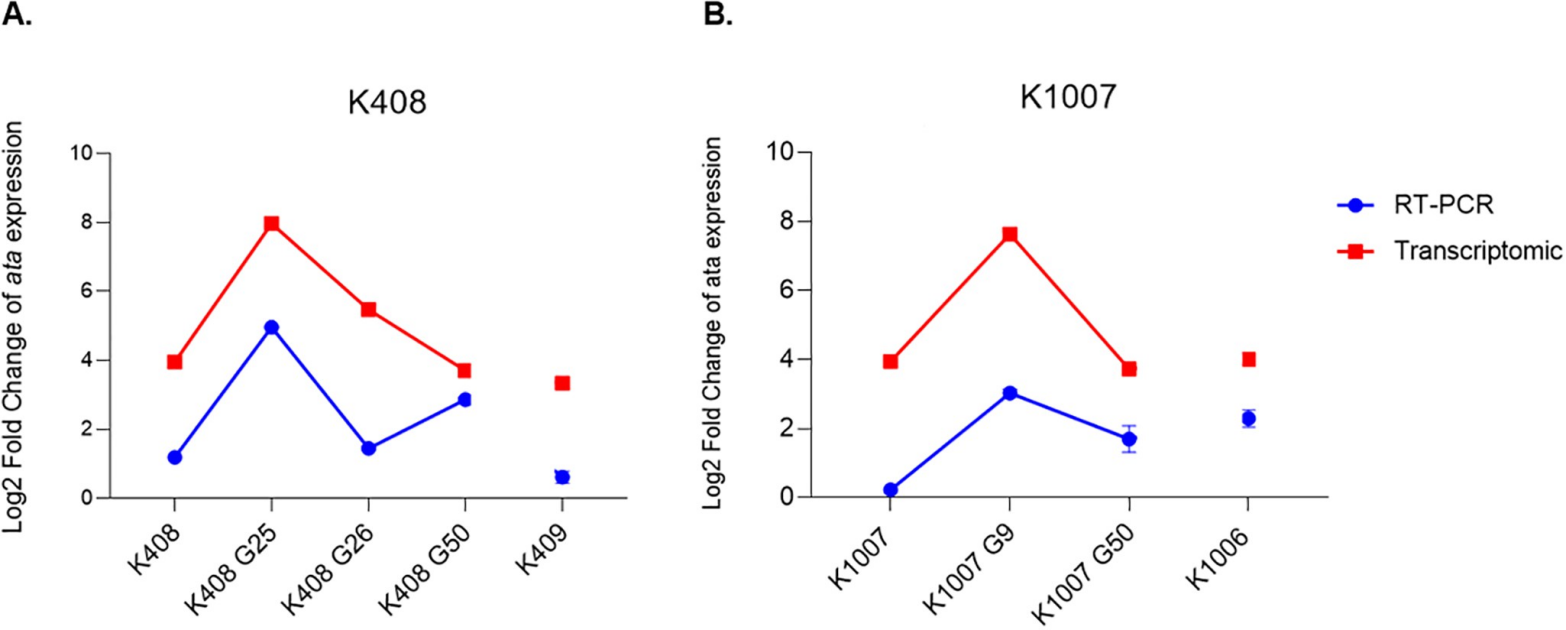
Transcriptomic and RT-PCR results (A) Gene expression levels of *ata* gene for the set of K408 cells, (B) Gene expression levels of *ata* gene for the set of K1007 cells.

The *ata* gene was highly upregulated on all *in vitro* colistin -exposed generations of the cell set of K408 (K408 G25, K408 G26, K408 G50). The *ata* gene expression of K408 G25 was 16.6 fold higher than K408 *(p* = 0.0004) while it was 11.4 fold in K408 G26 (Fig 3). In the set of K1007, although the ninth generation (K1007 G9) corresponding to clinical colistin resistance day was still colistin susceptible, expression of *ata* gene was 13.2 times higher compared to K1007 (*p* = 0.001). In the 50th generations of both cells, *ata* gene expression was downregulated to the level of susceptible (K408, K1007) and clinically resistant cells (K409, K1006).

### Serum resistance

**Fig 4:**
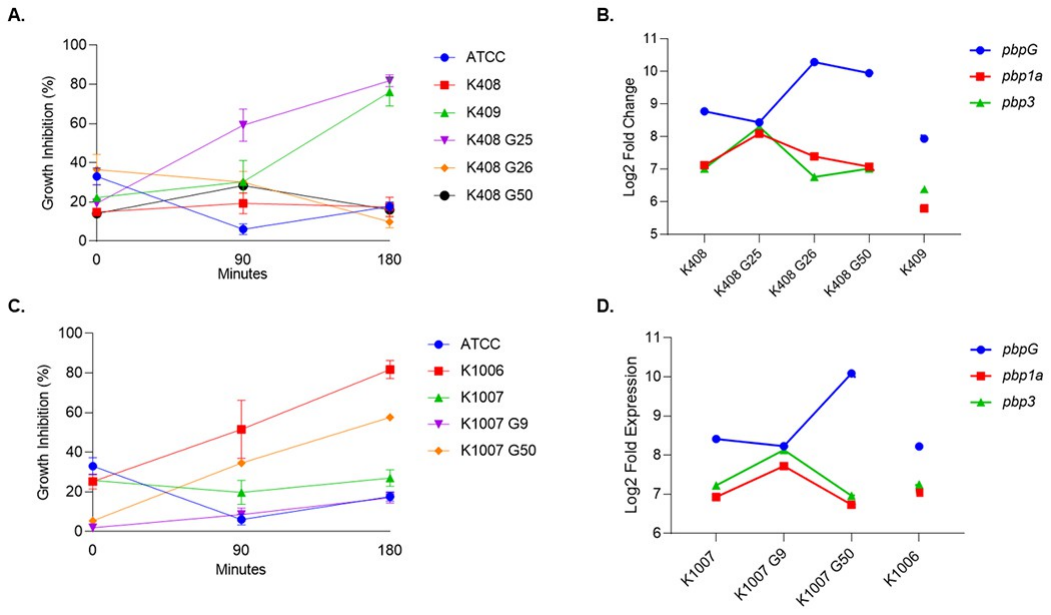
Growth inhibition results of serum resistance experiment. Calculated results using comparative incubation with inactivated human serum in (A) set of K408 cells (C) set of K1007 cells. Transcriptomic gene expression levels of serum resistance associated genes, *pbp3 (ftsl)*, *pbpG*, *pbp1a* in B) set of K408 cells and (D) set of K1007 cells.

Metagenomic analysis did not reveal any discriminative SNPs on serum resistance encoding genes. Transcriptomic analysis revealed that the *pbpG* starts to respond to colistin exposure after generations that corresponds to clinical colistin resistance days (K408 G25, K1007 G9) and a continuous increase was observed until the 50th generations of both sets of cells by 2.25 and 3.19 times higher than susceptible cells K408 and K1007, respectively (Fig 4). However, there was a controversial response from *pbp1* and *pbp3* to colistin exposure with a downregulation after generations that corresponded to clinical colistin resistance day (K408 G25, K1007 G9).

In the set of K408, a weak serum resistance was observed in colistin-resistant G25 with a 81.4% growth inhibition in human serum, followed by a recovery on the re-sensitized strain K408 G26 with a growth inhibition rate of 9.86% (*p* = 0.0018). Similarly in the set of K1007 cells, the growth inhibition rate of colistin susceptible K1007 was 26% while it was 78% in colistin resistant K1006 (Fig 4).

### RND efflux pumps

**Fig 5:**
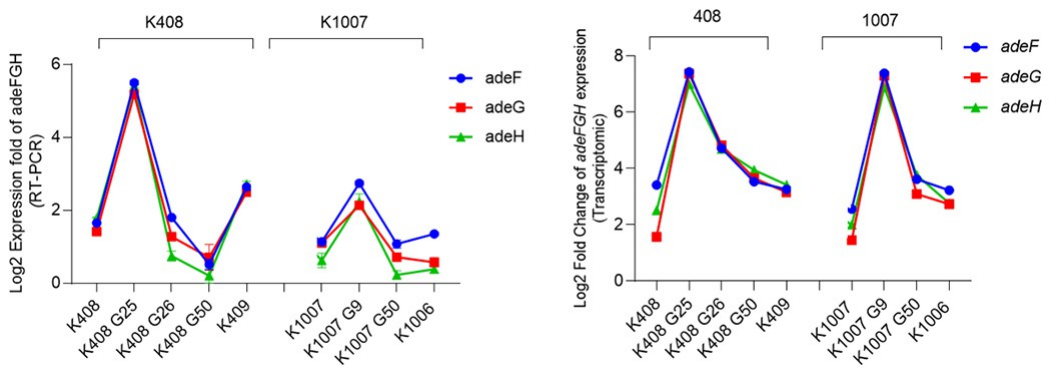
The adeFGH gene expression levels using transcriptomic results (A) in sets of 408 and 1007 cells obtained from transcriptome sequencing. The adeFGH gene expression levels using RT-PCR results (B) in sets of 408 and 1007 cells obtained from RT-PCR.

Among the RND efflux pump associated genes, the colistin exposure related changes were detected in *adeF*, *adeG*, *adeH* genes. All adeFGH efflux pump genes were upregulated in early stages of exposure until the clinical resistance corresponding day, followed by a downregulation upon prolonged exposure to colistin. The results were confirmed using qRT-PCR. The adeFGH expression was 5.2 and 2.1 times higher in K408 G25 and K1007G9 compared to K408 and 1007. (Fig 5).

There was no significant difference between clinically resistant and susceptible isolates (*p* = 0.2640). The gene expression results of *adeABC* and *adeIK* expressions were provided on S3 and S4 Figs.

### Outer membrane protein changes upon colistin exposure

**Fig 6:**
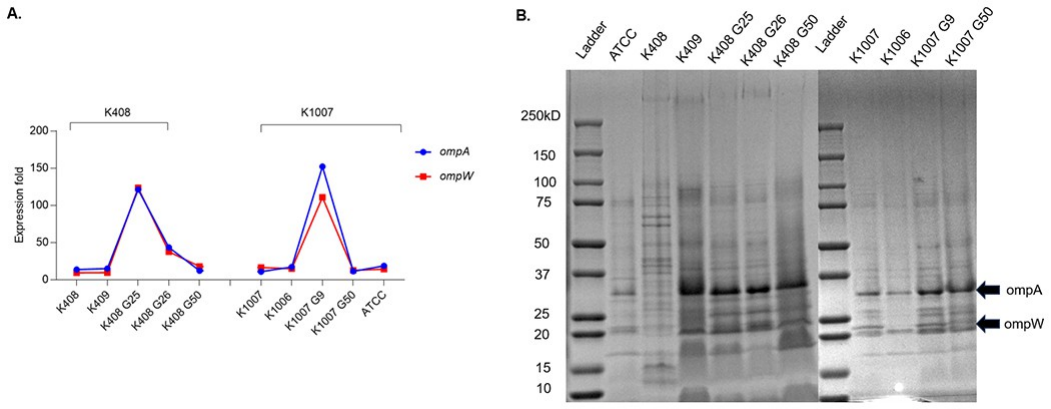
Transcriptomic gene expression results (A) Expression levels of *ompA* and *ompW* genes in sets of K408 and K1007 cells. SDS-PAGE results (B) Outer membrane proteins in sets of K408 and K1007 cells and *A*. *baumannii* ATCC 17978.

Transcriptomic analyses revealed upregulation of *ompW* gene by 9.3-fold and ompA by 13.6 fold in K408 G25 followed by downregulation of 2.81 and 3.33 fold upon resensitization to colistin (K408 G26) (Fig 6A). OmpW protein (~21 kDa) which was present in colistin susceptible K408 disappeared on K408 G25 and recovered in the resensitized K408 G26 (Fig 6B). In K1007 G9, *ompW* was upregulated 1.88. fold compared to K1007 and ompW protein was present. In this cell set, ompW protein was absent only in colistin resistant cell K1006.

Metagenomic analyses of *ompA* and *ompW* did not reveal any discriminative SNPs for both sets of cells (S1 Table).

**Fig 7:**
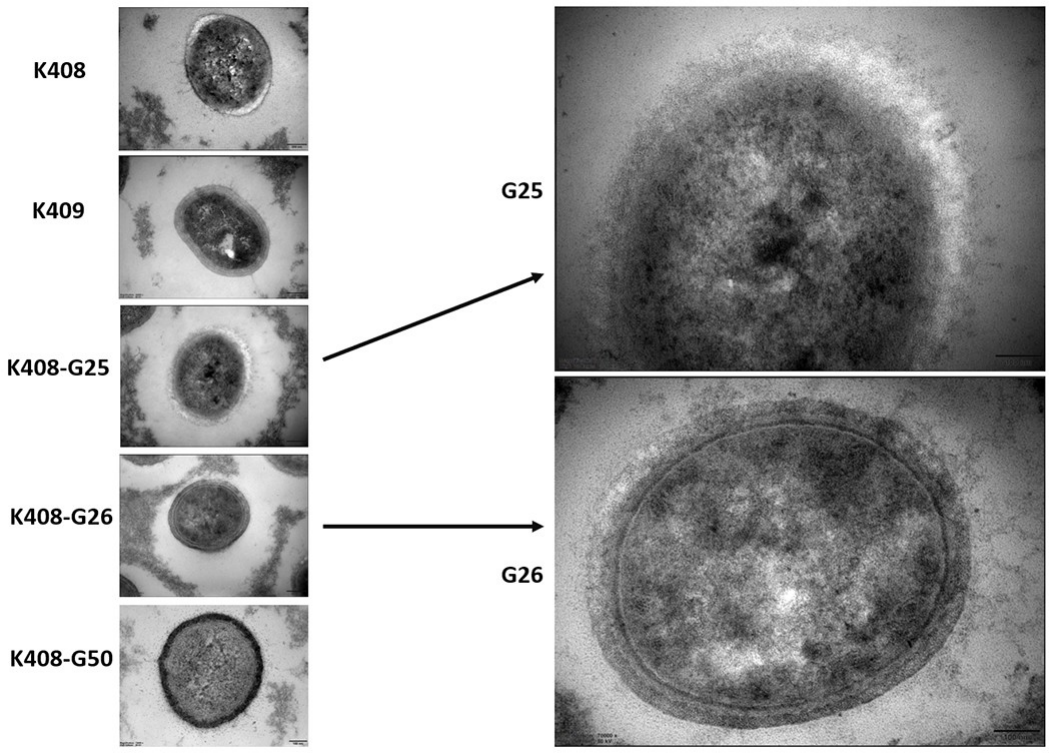
Transmission Electron Microscopy Imaging Sets of K408 cells under 40000X (left) and 70000X (right) magnification.

In the transmission electron microscopy analyses, a disrupted cell wall integrity and bleb formation was observed in K408 G25. In the resensitized generation, these pathological changes were fully recovered (Fig 7). In the cell set of K1007, no discriminative changes amongst cells were observed (S5 Fig).

Images of multiple cells on lower magnification (10,000X) are provided on S6 and S7 Figs.

### Biofilm production

**Fig 8:**
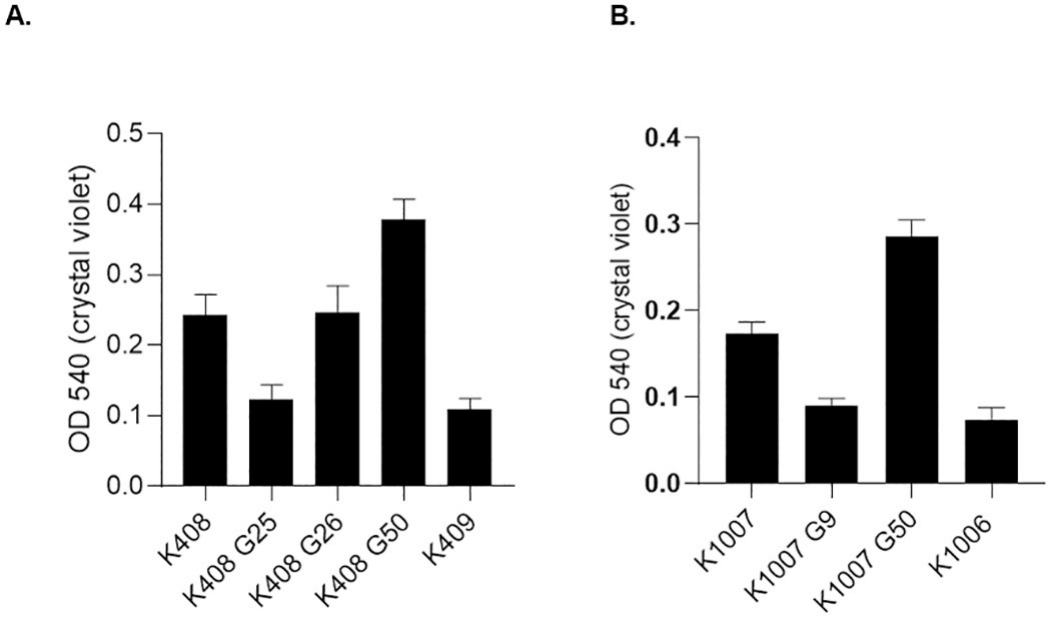
Biofilm production assay results at OD540 optical density measurement (A) et of K408 cells and (B) set of K1007 cells.

Lower biofilm production amongst colistin resistant cells (K408 G25, K409, K1006) was observed compared to colistin susceptible isolates with no statistical significance (*p* = 0.24). However, after the generations correspond to clinical resistance, the cells developed an adaptation to colistin exposure with enhanced biofilm production (Fig 8). The OD600 result of control strain *A*. *baumannii* ATCC 17978 was 0.144 (0.013±SE). Analysis of biofilm associated genes *bfmS*, *bfmR* had no discriminative SNPs in ColR isolates, additionally, expression levels of biofilm associated genes were not significantly differentiated amongst colistin susceptible and resistant isolates (S8 Fig).

## Discussion

Colistin resistance in *A*. *baumannii* is emerging despite the high biological cost of colistin stress to bacterium. Relatively low resistance rates reported for colistin resistance may be related to low use of colistin or poor adaptivity of *A*. *baumannii* to this antimicrobial upon exposure, however, increasing resistance rate is a concern [27]. Aydin et al. has reported colistin resistance rate to increase from 2% to 7% in Türkiye for *A*.*baumannii* from 2015 to 2018 [6]. In this study we investigated the stress response and adaptation mechanisms of *A*. *baumannii* under prolonged *in vitro* colistin exposure in parallel with paired clinical colistin susceptible and colistin resistant *A*. *baumannii* isolates.

The importance of this study is comparative assessment of virulence traits in clinically induced colistin resistance and *in vitro* induced colistin resistance on a phenotypic, metagenomic and transcriptomic basis. We also evaluated recovery and adaptation strategies in colistin-resensitized cells.

Amongst the cell sets, one of the colistin susceptible *A*. *baumannii* (K408) gained colistin resistance during *in vitro* induction on the corresponding day to clinical colistin resistance (25th day). However, K408 G25 got resensitized to colistin on the consecutive generation (K408 G26). The other isolate (K1007), colistin resistance did not develop amongst 50 generations of colistin induction. Previous studies reported that duration of colistin therapy differs amongst *A*. *baumannii* strains [28]. Our results confirm that colistin resistance development is a strain dependent behaviour with the contribution of additional *in vivo and* environmental stress factors.

We compared resistance and virulence associated genes such as *lpxA*, *lpxC*, *pmrA*, *pmrB*, *adeF*, *adeG*, *adeH*, *ompA*, *ompW*, *pbp7/8*, *pbp1a and pbp3*, *ata* in compliance with previously published studies on genomic and transcriptomic basis [7,29,30]. We detected significantly lower serum resistance activity in colistin resistant cells than colistin susceptible cells in both sets of cells. Decreased serum resistance is an important biological cost of *A*. *baumannii* upon conferring colistin resistance [31,32]. The serum resistance was significantly recovered in the colistin resensitized generation of K408 G26, while we observed weak resistance to serum in colistin resistant K408 G25 (*p* = 0.0018). In parallel with the recovery of serum resistance, *pbpG* gene was upregulated in the resensitized generation (G26) compared to G25. In contrast, pbp1a and pbp3 genes were downregulated in the same period. A similar pattern of response was observed in the K1007 set of cells as well. Previous studies reported that pbp genes interchangeably play an active role in peptidoglycan synthesis, permeability adaptation upon stress, conferring resistance to complement and interaction with septal sites during division [33–35]. In addition to this knowledge, we demonstrated a controversial activity of between *pbpG* and *pbp1/pbp3* genes in response to the colistin exposure. We also detected upregulation of ompA and ompW gene expressions in K408 G25 followed by a downregulation in the resensitized generation (K408 G26). Moreover, even though no colistin resistance was acquired during *in vitro* induction, K1007 cells displayed a similar trend on pbp, ompA and ompW gene expression. The controversial activity of the *ompA* and *ompW* genes to *pbpG* during developing resistance to cell wall targeted antibiotics was reported previously reported [36,37]. Our results show that although colistin is a cell membrane targeted antibiotic, *A*. *baumannii* possibly uses the same regulatory mechanisms for adaptation to colistin.

We observed loss of integrity in the cell wall of K408 G25 and recovery in the resensitized generation (G26). In previous studies, it was shown that colistin resistance leads to complete loss of LPS membrane [38]. Indeed, we observed that the outer membrane protein ompW disappeared in the K408 G25 but reappeared in the K408 G26. The recovery in the cell wall structure in our resensitized generation indicates the prompt structural response of the outer membrane of *A*. *baumannii* in the adaptation process. To support our postulation, we observed a disrupted cell wall integrity and exosomal bleb formation in K408 G25 which recovered in the following resensitized generation (K408 G26). The ompW was associated with escaping the complement cascade, enhancing resistance to phagocytosis and colistin binding [39–41]. Additionally, ompW protein is reported to be highly preserved and immunogenic while ΔompW strains are reported to be less virulent in terms of biofilm formation [42,43]. A recent study reported increased virulence scores of *A*. *baumannii* upon increased expression of *ompA* and *ompW* on persister cells of *A*. *baumannii* [44]. In our study, the reduced serum resistance ability on the colistin resistant cells (K408 G25, K409, K1006) which all lack the outer membrane ompW, supports the previous findings. Outer membrane proteins of *A*. *baumannii* is associated with potential therapeutic targets for drug and vaccination development research [45,46]. Our data suggests that ompW could be an antivirulence target for colistin susceptible *A*. *baumannii*, but it may not be appropriate for the prevention or treatment of colistin resistant *A*. *baumannii* infections.

Upregulation of RND efflux pump component adeFGH is associated with conferring antimicrobial resistance to *A*. *baumannii* [47]. In our study, we observed an increased gene expression of adeFGH in K408 G25 with a significant downregulation right after the resensitization of K408 G26 (*p* = 0.0022). Parallel behaviour was observed for the set of K1007 cells, as well. There were no discriminatory SNPs for RND efflux pump genes upon conferring colistin resistance. Significant downregulation of adeFGH was observed upon total recovery of the cells in G26 which hints that, RND efflux pumps plays a role in reducing the colistin stress of *A*. *baumannii* up until a chromosomal resistance is conferred. To support our hypothesis, we did not detect any SNPs in *in vitro* induced colistin resistance cells while clinically resistant cells (K409 and K1006) had SNPs on *pmrA* and *pmrB* which were also previously reported [48]. The upregulation of adeFGH in colistin resistant cells with intact *pmrB* was reported [49,50]. In addition to these reports, the downregulation of these genes in the resensitized generation of our study showed us that activation of RND efflux pumps is a biological cost in the absence of chromosomal resistance.

In the same trend to other virulence genes, *ata* gene expression was predominantly high in K408 G25 with a downregulation in K408 G26. It is stated that *ata* gene is associated with adherence and invasion of epithelial tissue, however, there is no published data on the change in *ata* gene upon colistin exposure while on the other hand previous studies reported that colistin resistance is related to lowered adherence [30]. We did not study a phenotypic adhesion assay however, based on our results we can postulate that *ata* expression is negatively correlated with adherence. The biofilm assay results were comparable with reduced adhesion upon acquiring colistin resistance. We observed that biofilm production was the lowest in K408 G25 and K1007 G9 followed by an increase towards 50th generations of colistin exposure.

Limitation of this study can be stated as the reflection of the *in vitro* experimental conditions *to in vivo* conditions. Although we tried to mimic the *in vivo* environment on *in vitro* colistin induction and serum resistance assay experiments, we could not mimic an intact immune system as this study did not involve animal studies.

## Conclusion

In this study we showed that, during the early stages of response to colistin stress until the development of chromosomal resistance, *Acinetobacter baumannii* regulates adhesion, efflux pump and serum resistance associated genes. Furthermore, the ompW protein disappears in the cell membrane of colistin resistant cells which recovers after resensitization to colistin. The disappearance of ompW in colistin resistant cells suggested that ompW targeted immunisation strategies could cause failure in prevention of colistin resistant *A*. *baumannii* infections.

## Supporting information

S1 Table Non-discriminative single nucleotide polymorphism results on both set of cells in K408 and K1007. (DOCX)

S2 Table List of oligonucleotide sequences used for qRT-PCR. (DOCX)

S1 Fig Dendrogram illustration for clonality analysis. Pulsed field gel electrophoresis results of K408, K409, K1006 and K1007 on assessment of clonal relatedness. Analysis was done using Bionumerics 7.6. (Biomerieux, France).
(TIF)

S2 Fig Heatmap illustration of transcriptomic data. Gene expression results of various virulence associated genes of *A*. *baumannii* based on transcriptomic data.
(TIF)

S3 Fig RT-PCR gene expression levels. Expression levels of *adeA*, *adeB*, *adeC*, *adeI* and *adeK* genes for the set of K408 cells.
(TIF)

S4 Fig RT-PCR gene expression levels. Expression levels of *adeA*, *adeB*, *adeC*, *adeI* and *adeK* genes for the set of K1007 cells.
(TIF)

S5 Fig Transmission electron microscopy imaging. Imaging of set of K1007 cells under 40000X (left) magnification and 70000X magnification (right).
(TIF)

S6 Fig Transmission electron microscopy imaging. Imaging of K408 set of cells under 10000X magnification.
(TIF)

S7 Fig Transmission electron microscopy imaging. Imaging of K1007 set of cells under 10000X magnification.
(TIF)

S8 Fig Gene expression levels based on transcriptomic data. Expression levels of biofilm associated genes for sets of K1007 and K408 cells.
(TIF)
