# Supplementary material for: Resensitization to colistin results in rapid and stable recovery of adherence, serum resistance and ompW in Acinetobacter baumannii
Source: PLoS One. 2024 Aug 28;19(8):e0309307. doi: 10.1371/journal.pone.0309307 (PMC11356438; doi:10.1371/journal.pone.0309307)
Supplement: S1 Table — (DOCX) [file pone.0309307.s001.docx]

S1 Table . Non-discriminative single nucleotide polymorphism results on both set of cells in K408 and K1007.

| **Function** | **Gene** | **Annotation Type** | **Codon Change** | **Protein Change** | **Isolate Codes** |  |  |
| --- | --- | --- | --- | --- | --- | --- | --- |
|  |  |  |  |  |  |  |  |
| **Adhesion** | *ata* | SNP | 2676C>G | As92Glu | K409, K408, K408 G25, K408 G26, K1007, K1007 G50 |  |  |
|  | *ata* | SNP | 4065T>A | As355Glu | K409, K408, K408 G25, K408 G26, K408 G50, K1006 K1007, K1007 G50 |  |  |
|  | *ata* | SNP | 4086T>A | As362Glu | K408 G25 |  |  |
|  | *ata* | SNP | 4117G>A | As373Asn | K408, K408 G25, K408 G26, K409, K1006, K1007 G50 |  |  |
|  | *ata* | SNP | 1237C>A | Arg413Ser | K1007, K1007 G9, K1007 G50 |  |  |
|  | *ata* | SNP | 2597A>G | Asn866Ser | K408 G25, K409, K1007 G50 |  |  |
|  | *ata* | SNP | 2717C>T | Ala906Val | K408 G25, K409, K1007, K1007 G50 |  |  |
|  | *ata* | SNP | 3925G>A | Gly1309Ser | K1007, K1007 G9, K1007 G50 |  |  |
|  | *ata* | SNP | 4126A>G | Asn1376Asp | K408, K408 G25, K408 G26, K409, K1006, K1007 G50 |  |  |
| **RND Efflux pump** | *adeA* | SNP | 605A>T | Gln202Leu | K1007, K1007 G9, K1007 G50 |  |  |
| **Serum resistance** | *ftsI* | SNP | 1544C>T | Ala515Val | K1006 |  |  |
| **Quorum Sensing** | *abaR* | SNP | 69G>T | Glu23Asp | K1007, K1007 G9, K1007 G50 |  |  |
|  | *abaR* | SNP | 205C>G | Arg69Gly | K1007, K1007 G9, K1007 G50 |  |  |
|  | *abaI* | SNP | 133A>G | Asn45Asp | K1007, K1007 G9, K1007 G50 |  |  |
|  | *abaI* | SNP | 160G>A | Val54Ile | K408, K408 G25, K408 G26, K409, K408 G50 |  |  |
|  | *abaI* | SNP | 282G>A | Met94Ile | K1007, K1007 G9, K1007 G50 |  |  |
|  | *abaI* | SNP | 359G>A | Ser120Asn | K1006 |  |  |
|  | *abaI* | SNP | 358A>G | Ser120Gly | K1007, K1007 G9, K1007 G50 |  |  |
|  | *abaI* | SNP | 359G>C | Ser120Thr | K1007, K1007 G9, K1007 G50 |  |  |
