# Supplementary material for: Resensitization to colistin results in rapid and stable recovery of adherence, serum resistance and ompW in Acinetobacter baumannii
Source: PLoS One. 2024 Aug 28;19(8):e0309307. doi: 10.1371/journal.pone.0309307 (PMC11356438; doi:10.1371/journal.pone.0309307)
Supplement: S2 Table — (DOCX) [file pone.0309307.s002.docx]

S2 Table. List of oligonucleotide sequences used for qRT-PCR

| Oligonucleotide Name | Sequence (5-'3') | Source |
| --- | --- | --- |
| CarO-RT-F | GGCGGATGAAGCTGTTGTTC | Fernandez Cuenca et al 2015 |
| CarO-RT-R | CACGAGCGCCTACTGGAATT |  |
| OmpA-RT-F | AGCTCTTGCTGGCTTAAACG |  |
| OmpA-RT-R | GAGCAACTGGAGTTGGTTCA |  |
| 16s rRNA-F | TCAGCTCGTGTCGTGAGATG | Beceiro et al 2011 |
| 16s rRNA-R | CGTAAGGGCCATGATG |  |
| adeF-F | GGTGTCGACCAAGATAAACG | Coyne et al 2009 |
| adeF-R | GTGAATTTGGCATAGGGACG |  |
| adeG-F | GTGTAGTGCCACTGGTTACT |  |
| adeG-R | ATGTGGGCTAGCTAACGGC |  |
| adeH-F | CGATCAGCAAATTCAGGCTC |  |
| adeH-R | GCTTGCAATGATTTGGCTGC |  |
| adeA-F | ATCGCTAACAAAGGCTTGAA |  |
| adeA-R | CGCCCCCTCAGCTATAGAA |  |
| adeB-F | CTTGCATTTACGTGTGGTGT |  |
| adeB-R | GCTTTTCTACTGCACCCAAA |  |
| adeC-F | TACACATGCGCATATTGGTG |  |
| adeC-R | CGTAAAATAACTATCCACTCC |  |
| adeI-F | CAAATGCAAATGTAGATCTTGG |  |
| adeI-R | AAACTGCCTTTACTTAGTTG |  |
| adeJ-F | GGTCATTAATATCTTTGGC |  |
| adeJ-R | GGTACGAATACCGCTGTCA |  |
| adeK-F | TTGATAGTTACTTGACTGTTC |  |
| adeK-R | GGTTGGTGAACCACTGTATC |  |
| pmrA-F | ATGACAAAAATCTTGATGATTGAAGAT | Beceiro et al 2011 |
| pmrA-R | CCATCATAGGCAATCCTAAATCCA |  |
| pmrB-F | GAACAGCTGAGCACCCTTTAA |  |
| pmrB-R | ACAGGTGGAACCAGCAAATG |  |
| pmrC-F | CTCTTTACGCTTTGTTTTATGGAC |  |
| pmrC-R | GTAAAAAGTAAAACACCGACCA |  |
| ata-F | ATTCGGTGCTGTTGCACAAG | Weidensdorfer et al 2018 |
| ata-R | CACCCGGTTTATTACCAGAG |  |
