# Supplementary figures and images for: Resensitization to colistin results in rapid and stable recovery of adherence, serum resistance and ompW in Acinetobacter baumannii
Source: PLoS One. 2024 Aug 28;19(8):e0309307. doi: 10.1371/journal.pone.0309307 (PMC11356438; doi:10.1371/journal.pone.0309307)

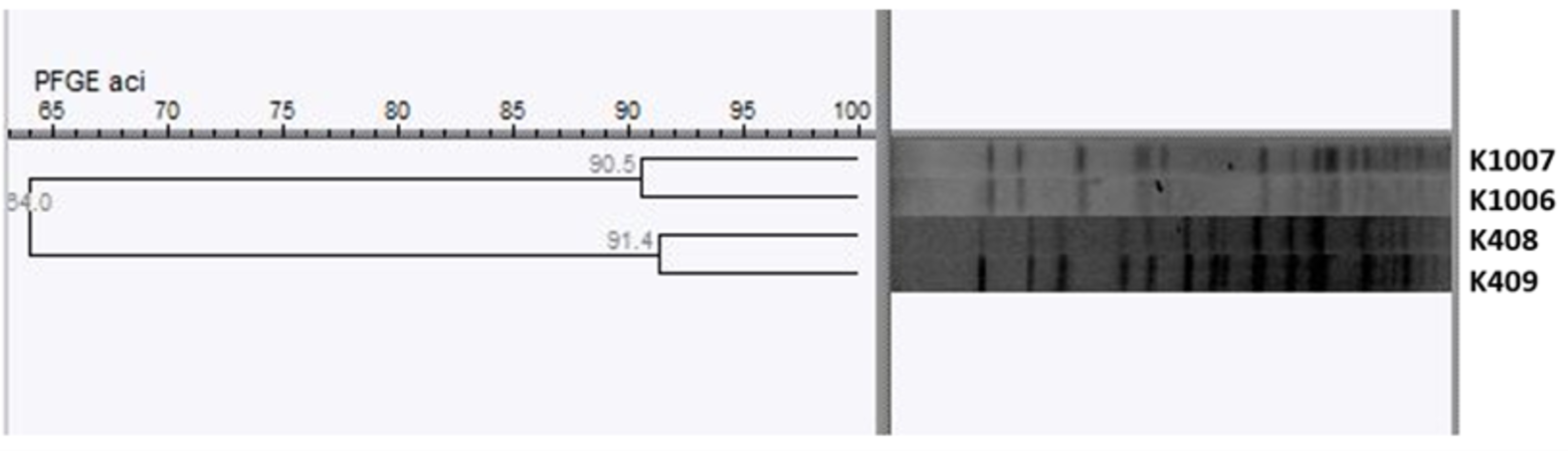

Supplement: S1 Fig — Pulsed field gel electrophoresis results of K408, K409, K1006 and K1007 on assessment of clonal relatedness. Analysis was done using Bionumerics 7.6. (Biomerieux, France). (TIF) [file pone.0309307.s003.tif]

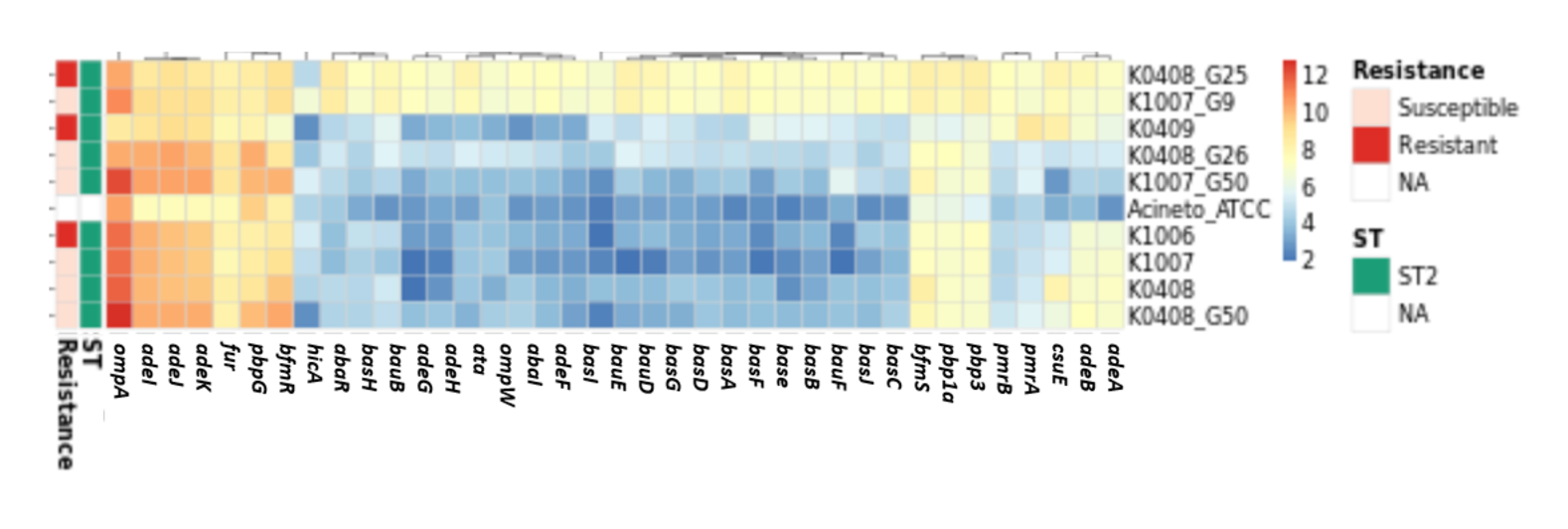

Supplement: S2 Fig — Gene expression results of various virulence associated genes of A. baumannii based on transcriptomic data. (TIF) [file pone.0309307.s004.tif]

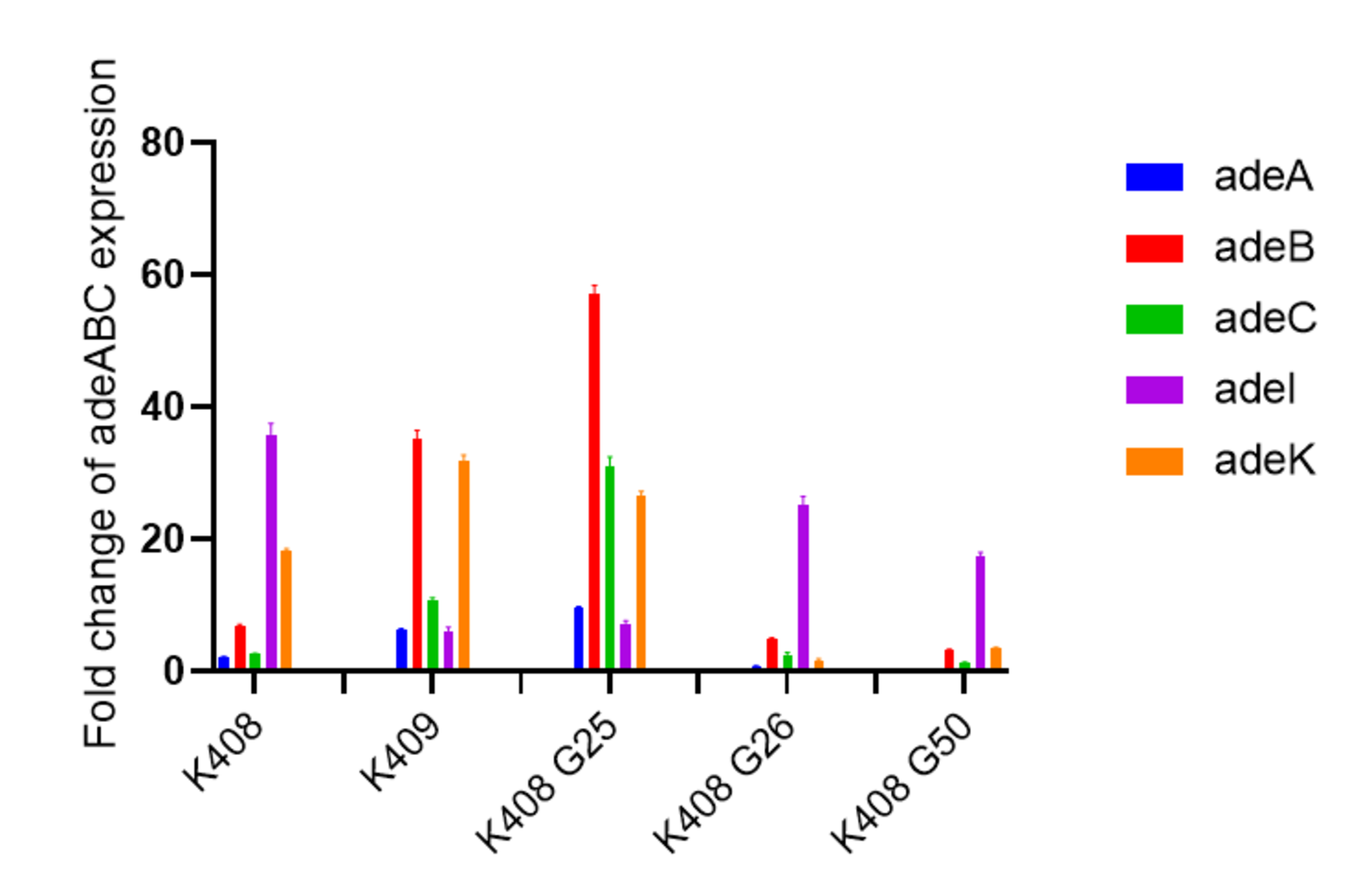

Supplement: S3 Fig — Expression levels of adeA, adeB, adeC, adeI and adeK genes for the set of K408 cells. (TIF) [file pone.0309307.s005.tif]

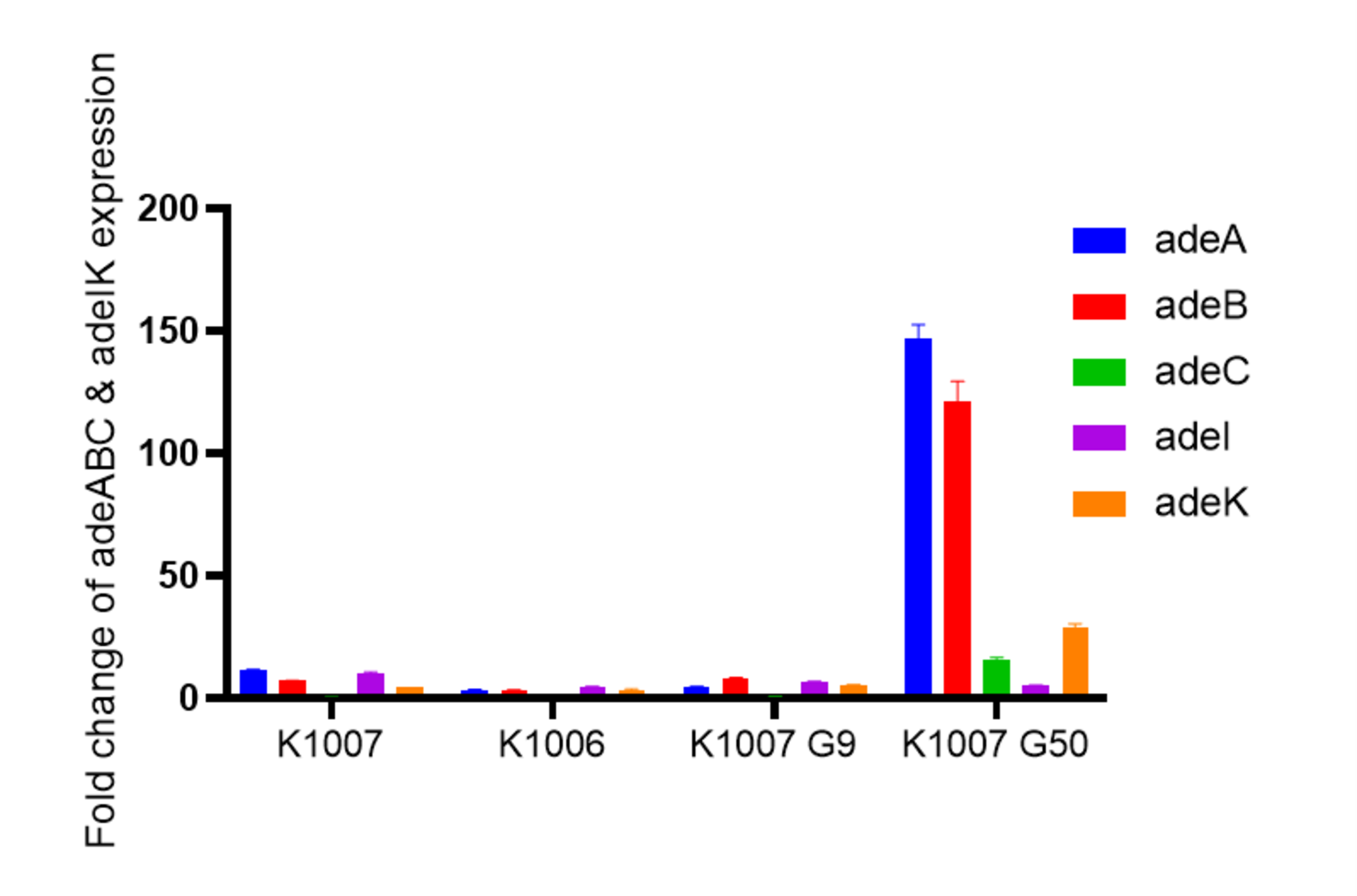

Supplement: S4 Fig — Expression levels of adeA, adeB, adeC, adeI and adeK genes for the set of K1007 cells. (TIF) [file pone.0309307.s006.tif]

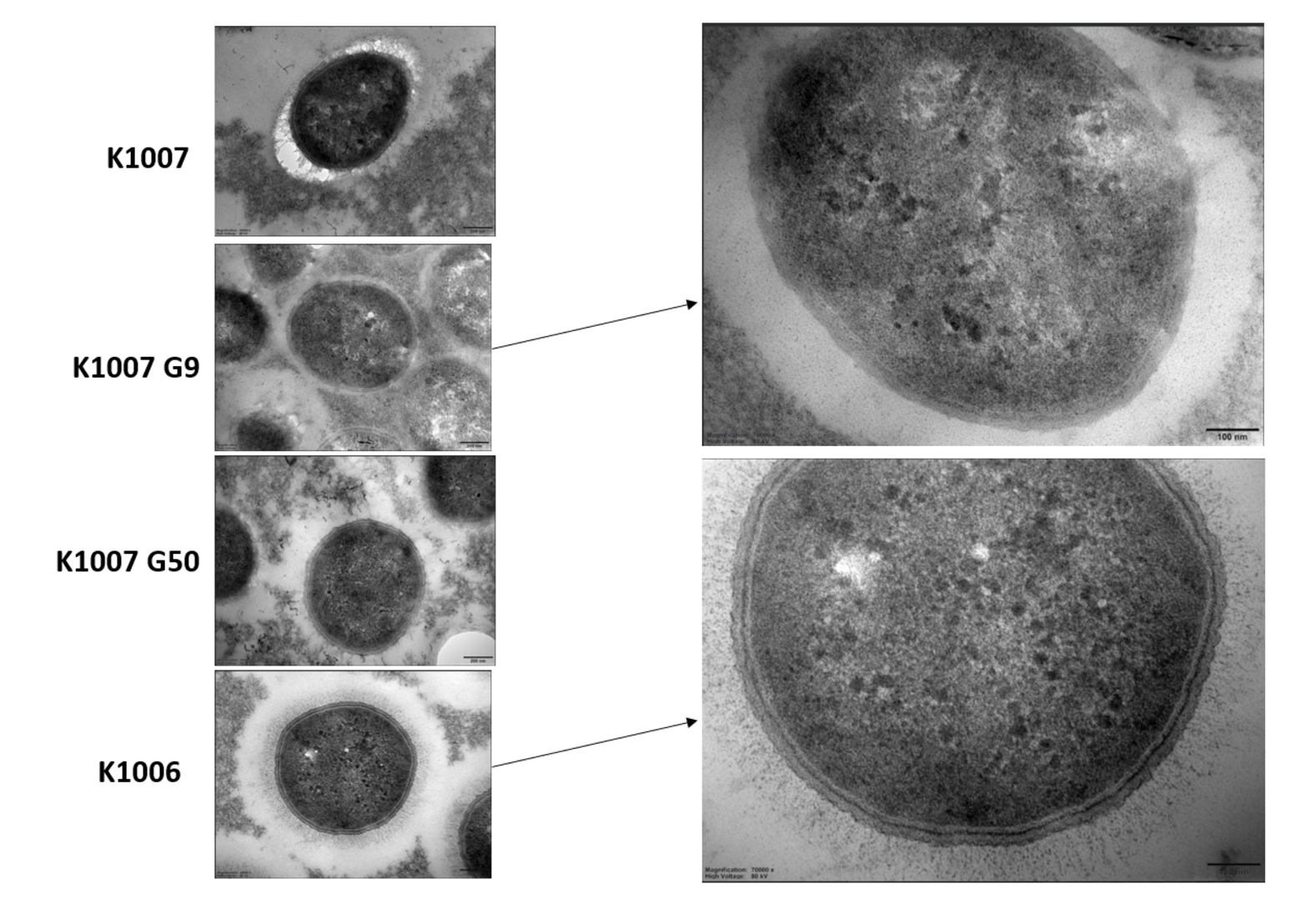

Supplement: S5 Fig — Imaging of set of K1007 cells under 40000X (left) magnification and 70000X magnification (right). (TIF) [file pone.0309307.s007.tif]

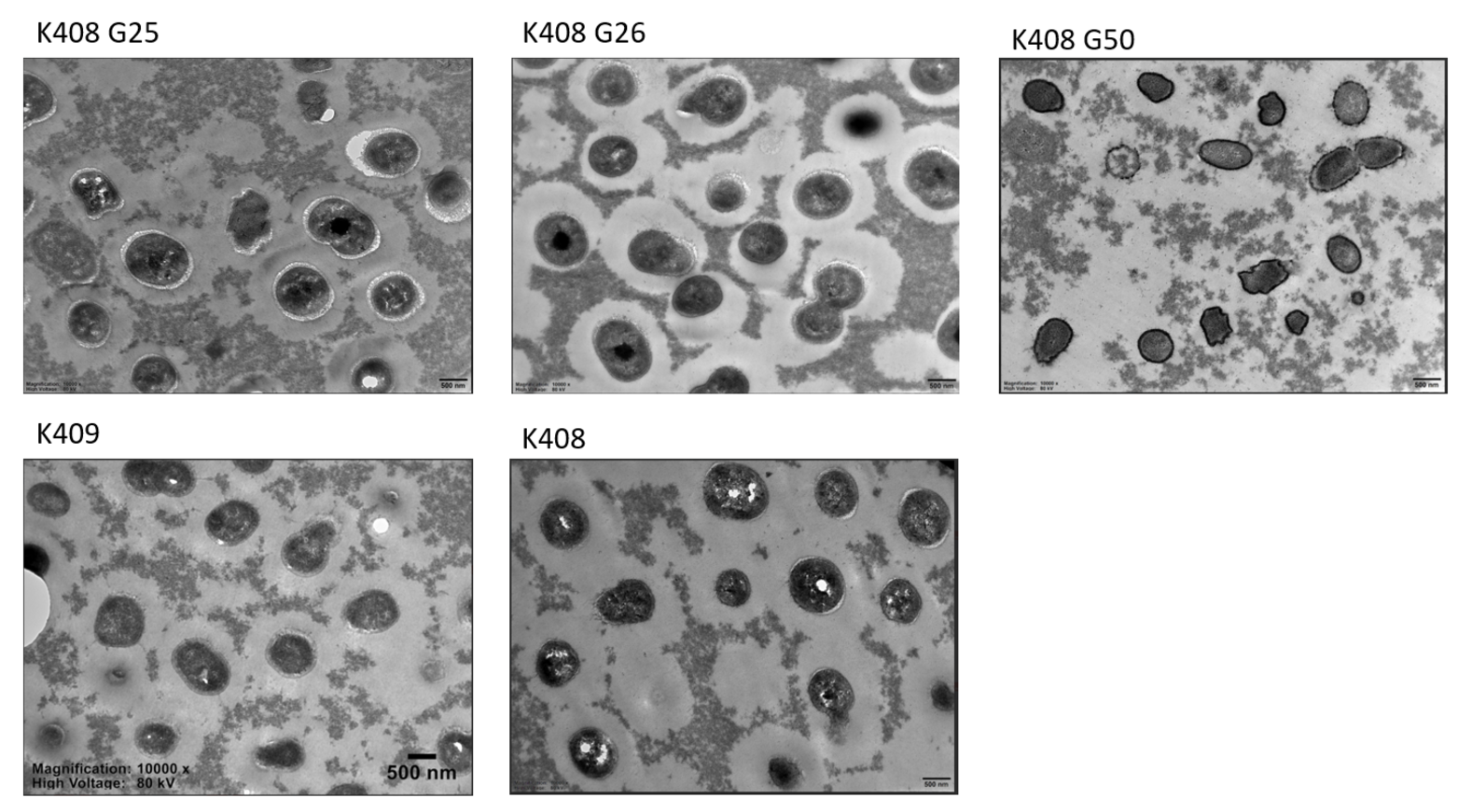

Supplement: S6 Fig — Imaging of K408 set of cells under 10000X magnification. (TIF) [file pone.0309307.s008.tif]

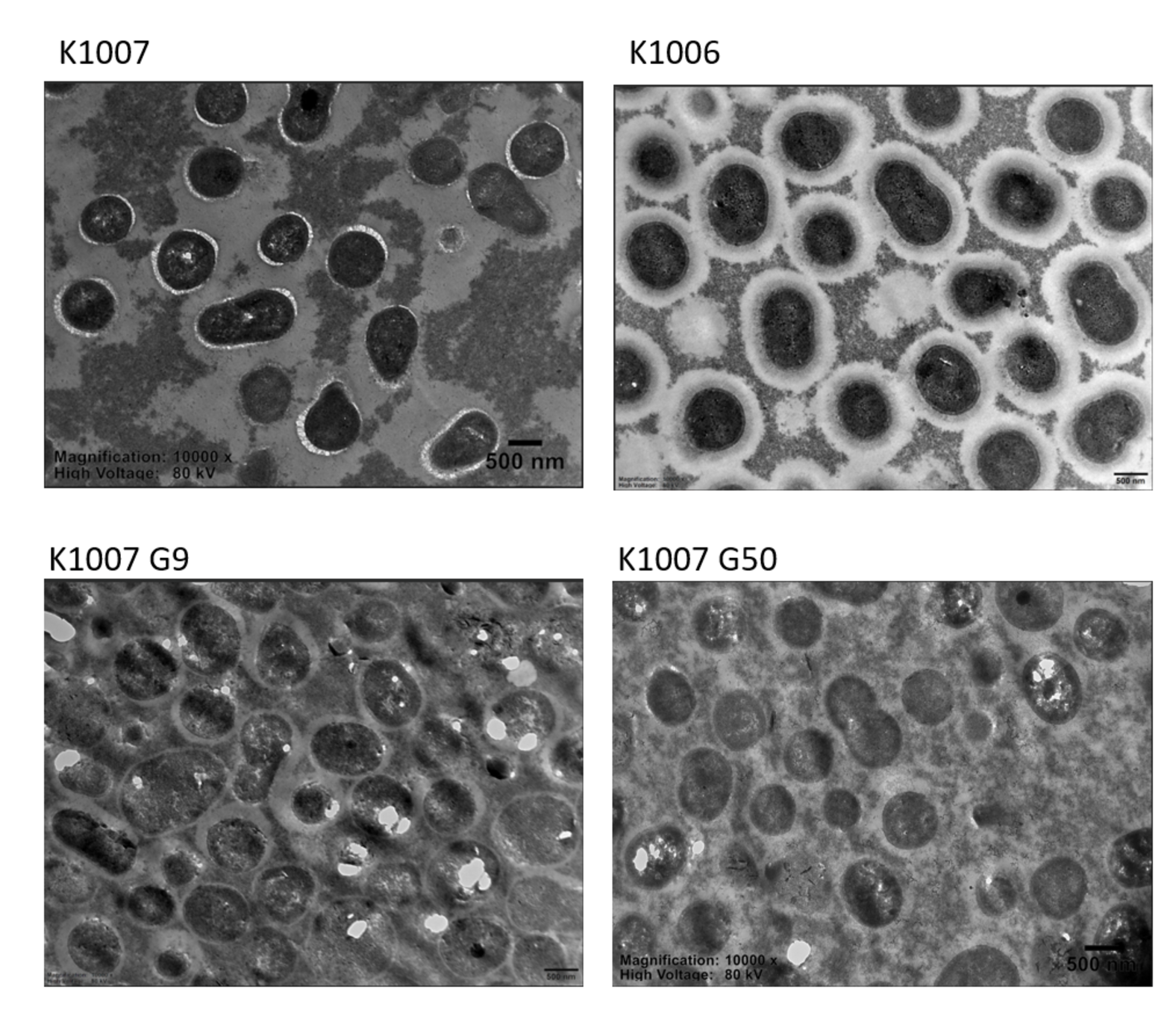

Supplement: S7 Fig — Imaging of K1007 set of cells under 10000X magnification. (TIF) [file pone.0309307.s009.tif]

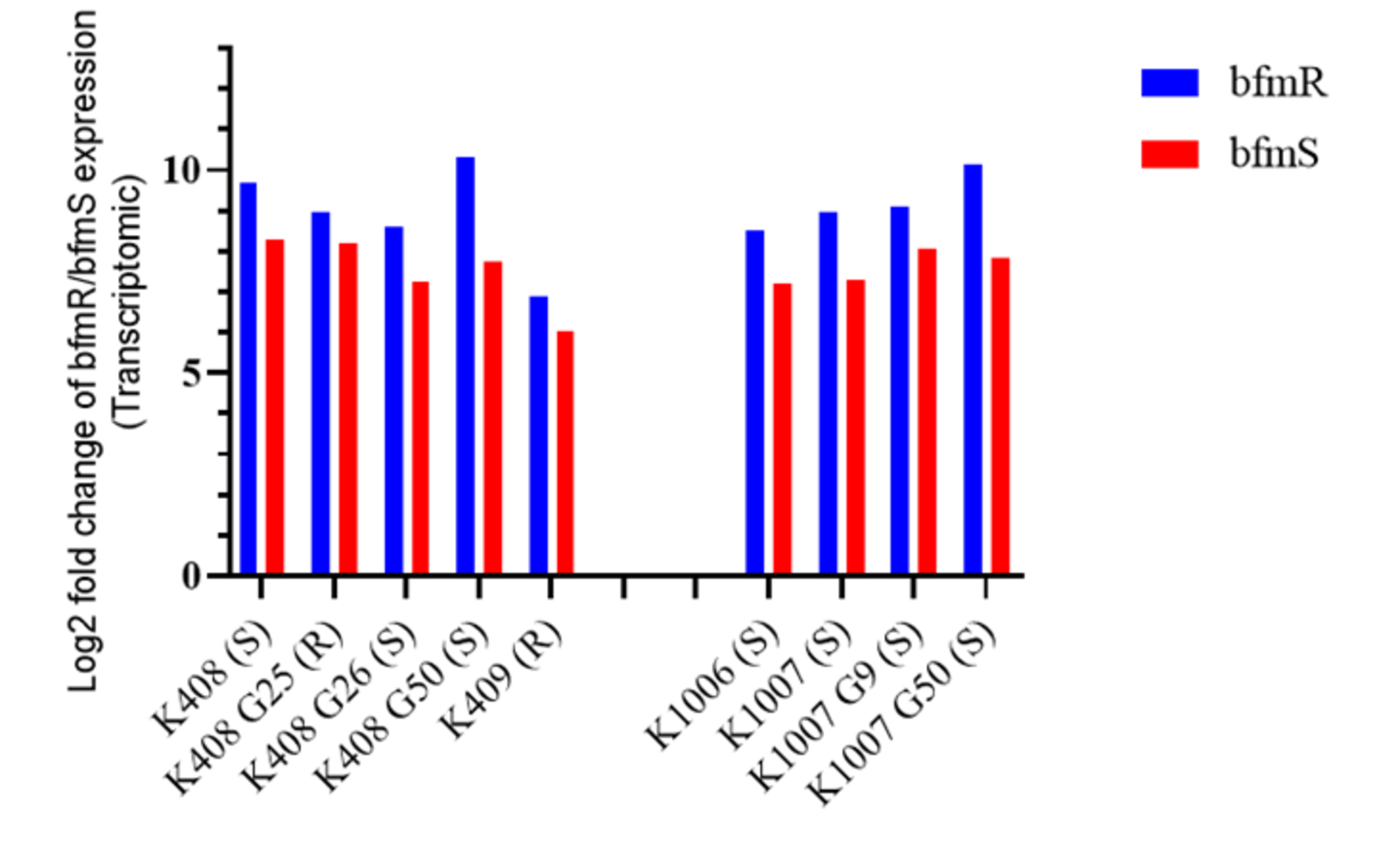

Supplement: S8 Fig — Expression levels of biofilm associated genes for sets of K1007 and K408 cells. (TIF) [file pone.0309307.s010.tif]
